# Niemann-Pick disease type-B: a unique case report with compound heterozygosity and complicated lipid management

**DOI:** 10.1186/s12881-020-01027-9

**Published:** 2020-05-06

**Authors:** L. Ordieres-Ortega, F. Galeano-Valle, M. Mallén-Pérez, C. Muñoz-Delgado, J. E. Apaza-Chavez, F. J. Menárguez-Palanca, L. A. Alvarez-Sala Walther, P. Demelo-Rodríguez

**Affiliations:** 1 Internal Medicine Department, Hospital General Universitario Gregorio Marañón, Calle Dr. Esquerdo 46, 28007 Madrid, Spain; 2 Department of Medicine. Facultad de Medicina, Universidad Complutense, Madrid, Spain; 3 Instituto de investigaciones Sanitarias Gregorio Marañón (IiSGM), Calle Doctor Esquerdo, 46 Madrid, Spain; 4 Departament of Biochemistry and Molecular and Cellular Biology. Facultad de Ciencias, Universidad de Zaragoza, Calle Menéndez Pelayo, 24, 50009 Zaragoza, Spain; 5 Rare Diseases Unit. Hospital General Universitario Gregorio Marañón, Calle Doctor Esquerdo 46, Madrid, Spain; 6 Department of Pathology, Hospital General Universitario Gregorio Marañón, Calle Doctor Esquerdo 46, Madrid, Spain

**Keywords:** Niemann-Pick disease, type B, Sphingomyelin phosphodiesterase, Low HDL cholesterol, Genetics

## Abstract

**Background:**

Niemann-Pick disease (NPD) is a rare autosomal recessive hereditary disease characterized by deficient activity of acid sphingomyelinase.

**Case presentation:**

We present a case of NPD type B with a unique compound heterozygosity for SMPD1 (NM_000543.4:c.[84delC];[96G > A]) in which both mutations that induce an early stop codon are located before the second in-frame initiation codon. The clinical presentation of the patient is compatible with NPD type B. She was initially diagnosed of Gaucher Disease, but her altered lipid profile led to a clinical suspicion of NPD.

Combined high doses of atorvastatin and ezetimibe were given to treat the severe hypercholesterolemia.

**Conclusions:**

The pharmacological management of the lipid profile in these patients is important. A unique compound mutation in SMPD1 gene is described.

## Background

Niemann-Pick disease (NPD) is a rare autosomal recessive hereditary disease without gender predilection, with an estimated frequency of 0.4–1 in 100,000 newborns [1], characterized by deficient activity of acid sphingomyelinase (ASM; E.C. 3.1.4.12). The accumulation of vacuolated lipid-filled macrophages, known as Niemann-Pick cells, leads to the involvement of different organs: liver, spleen, bone marrow, lung and central nervous system (CNS) [2].

NPD type A (MIM# 257200) is a neurodegenerative disorder with massive hepatosplenomegaly and rapidly progressive neurologic course, leading to death around the age of three. NPD type B (MIM# 607616) is a non-neuropathic disorder causing hepatosplenomegaly and pulmonary involvement. Patients usually reach adulthood and their CNS is unaffected. Both types A and B are caused by mutations in SMPD1 gene, unlike type C, which is caused by mutations in NPC1 and NPC2 genes. Diagnosis is performed by bone marrow (BM) biopsies revealing accumulation of lipid- filled macrophages, known as sea-blue histiocytes [2], or the demonstration of low sphingomyelinase activity in an enzyme assay of cultured skin fibroblasts or isolated leukocytes [1].

ASM or sphingomyelin phosphodiesterase-1 is encoded by the SMPD1 gene (MIM#607608, GenBank accession number M81780.1) [3]. More than 140 mutations in NPD have been reported (Human Gene Mutation Database: http://www.hgmd.org). All diagnosed patients with symptoms and low ASM activity should get a genetic analysis, as prognosis, and even therapeutic management can differ. No specific treatment is currently available. New therapies, such as olipudase, are under research [1].

We present a case of NPD type B that has been previously reported within a series of NPD type A and B patients [4]. This is a unique case of compound heterozygosity in which both mutations induce a premature stop codon and both are located before the second in-frame initiation codon that is present in the SMPD1 gene. New information is added, as the genetic pedigree of her family and her clinical evolution 19 years later are presented. We review its rare genetic mechanism of action and the very scarce cases of lipid-lowering management of NPD in the literature.

## Case presentation

Our patient, a Caucasian female, was misdiagnosed with Gaucher disease at the age of two, due to mild hepatosplenic enlargement. Both liver and BM biopsies suggested this diagnosis, but these studies were done elsewhere and results were not available. She underwent treatment with steroids until the age of 18.

**Fig. 1:**
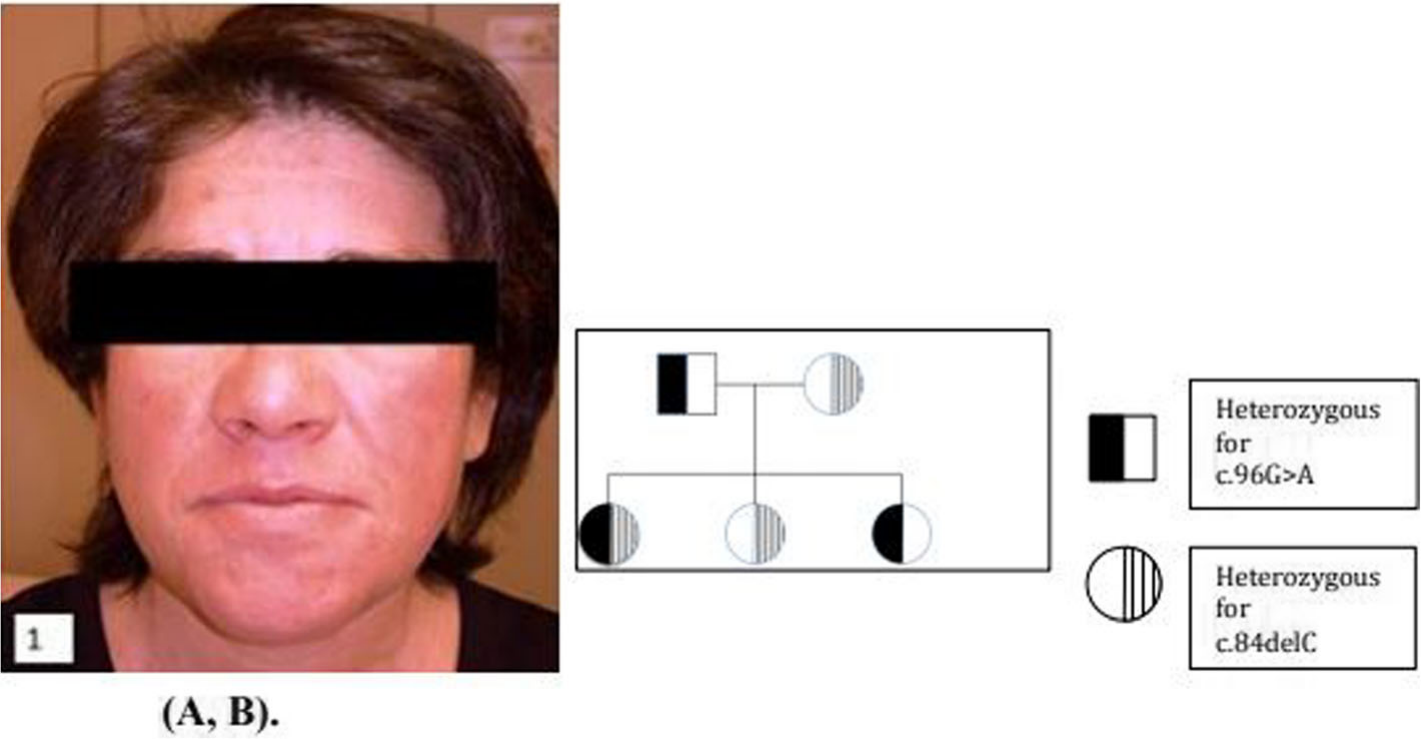
**a** Facial presentation showing a wide jaw, significantly closed palpebral fissure, and mildly violaceous color over the cheeks and forehead. Taken at the age of 55 years old. **b** Familial pedigree of the patient: 5429 Father: Nucleotide: NM_000543.4:c.[96G > A];[96=]; Protein: NP_000534.3:p.[(Trp32Ter)];[Trp32=]; rs3838786: five/six. 5430 Mother: NM_000543.4:c.[84delC];[84=]; NP_000534.3:p.[(Gly29AspfsTer48)];[Gly29=]; rs3838786: six/six. 5431 Index case: NM_000543.4:c.[84delC];[96G > A]; NP_000534.3:p.[(Gly29AspfsTer48)];[(Trp32Ter)]; rs3838786: six/six. 5432 Sister 1: NM_000543.4:c.[84delC];[84=]; NP_000534.3:p.[(Gly29AspfsTer48)];[Gly29=]; rs3838786: five/six. 5433 Sister 2: NM_000543.4:c.[96G > A];[96=]; NP_000534.3:p.[(Trp32Ter)];[Trp32=]; rs3838786: six/six

At the age of 36, she presented in Internal Medicine with a 2-year history of mild dyspnoea. She had previously been asymptomatic. Physical examination revealed a wide jaw, significantly closed palpebral fissures, due to upper palpebral thickening and mildly violaceous colour over her cheeks and forehead that had increased along the years (Fig. 1a).

**Fig. 2:**
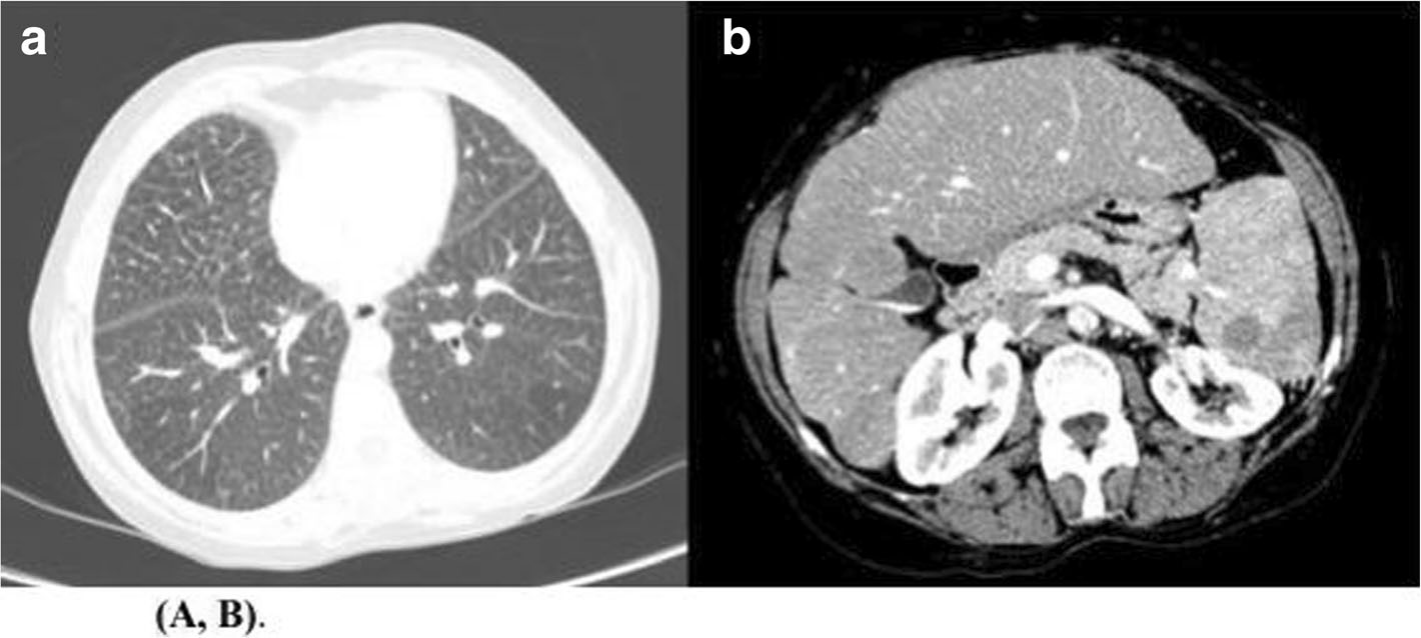
Computerized tomography (CT). **a.** Crazy-paven pattern of the lungs with calcified granulomas. **b.** Hepatic cirrhosis with hypodense nodes on the left hepatic lobe, signs of portal hypertension and splenic nodes

**Fig. 3:**
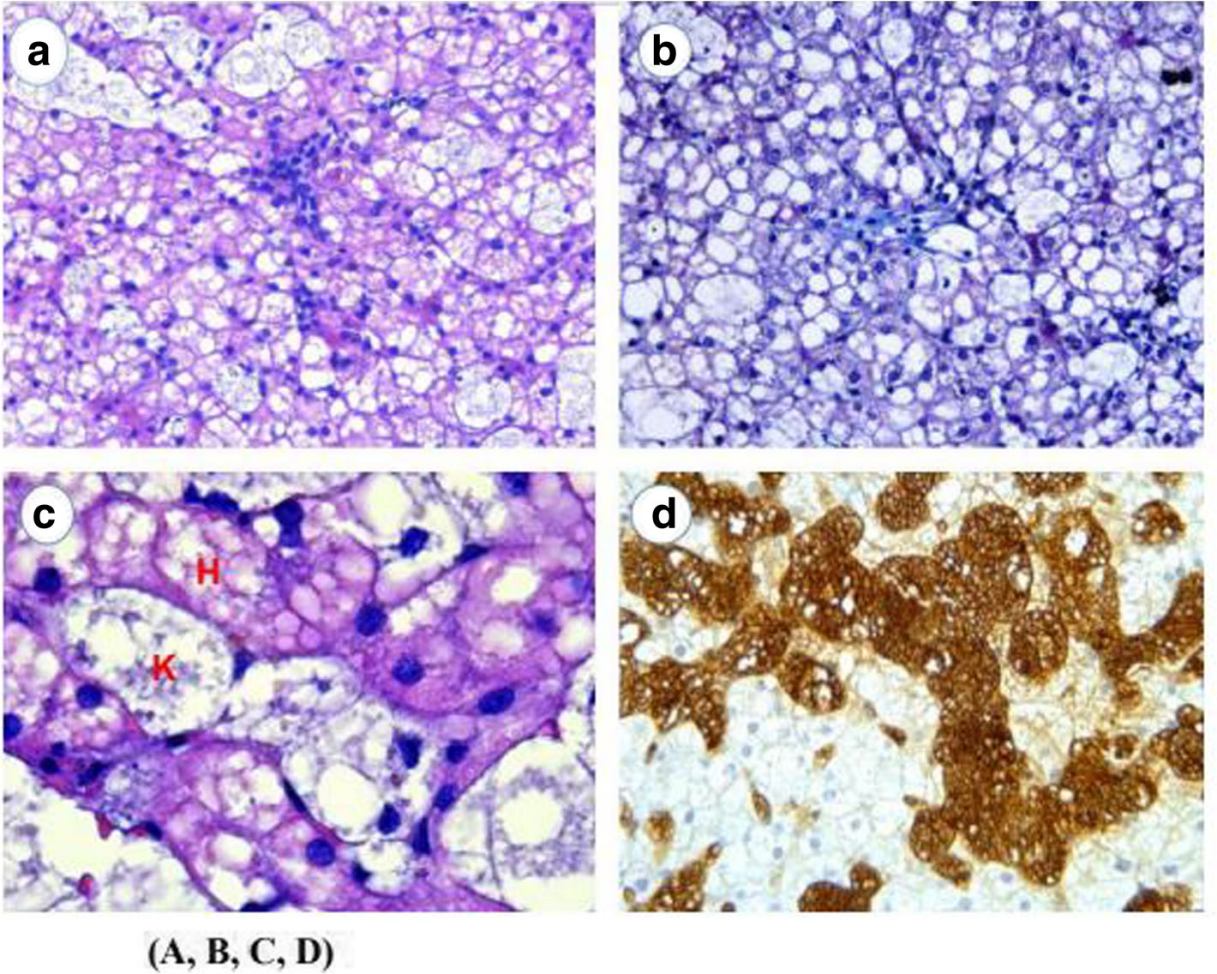
**a.** Liver biopsy demonstrates enlarged hepatocytes with a finely vacuolated cytoplasm and generalised foamy Kupffer cells filling the sinusoids (H-E staining x 200). **b.** Trichrome-stained sections show minimal perisinusoidal fibrosis (x 200). **c.** Higher magnification shows cytoplasmic vacuolization in hepatocytes (H), as well as in Kupffer cells (K) (H-E staining x 1000). **d.** Positive strong reactivity for CD68 highlights sinusoidal cells

Pulmonary auscultation showed audible pulmonary crackles. Abdominal examination demonstrated moderate hepatosplenomegaly. Blood tests showed a normal cell count, alkaline phosphatase 477 U/L, gamma-glutamyl transferase 53 U/L, alanine aminotransferase 102 U/L, aspartate aminotransferase 76 U/L, bilirubin 1 mg/dL, total cholesterol 445 mg/dL, triglycerides 287 mg/dL and high-density lipoproteins (HDL) cholesterol 16 mg/dL. An abdominal ultrasound disclosed an enlarged liver with hyper echogenic images compatible with cholesterol deposits. A liver biopsy done at that age revealed finely vacuolated hepatocytes and Kupffer cells and slight congestion of the central vessels, suggestive of a metabolic condition (Fig. 2). A BM aspirate in the same year showed presence of sea-blue histiocytes, leading to the suspicion of NPD, along with the altered lipid profile. An electrocardiogram, a spirometry and an ophthalmologic examination were normal. A chest X-ray evidenced an interstitial bilateral infiltrate. At the age of 44, a computerized tomography (CT) (Fig. 3) revealed a crazy-paving pattern of the lungs, with calcified granulomas, and hepatic cirrhosis with hypodense nodes on the left hepatic lobe, signs of portal hypertension and splenic nodes. At the 36 years old visit, an enzyme assay measuring ASM activity in leukocytes showed values of 31 nmol/mg min vs. 692.5 nmol/mg min in a control, meaning a 4.5% of activity, establishing the diagnosis of NPD. The analysis of the *SMPD1* gene revealed the presence of a double heterozygous genetic defect: NM_000543.4:c.[84delC];[96G > A]. The genetic pedigree of her family is indicated as follows (Fig. 1b), all of them being asymptomatic.

**Fig. 4:**
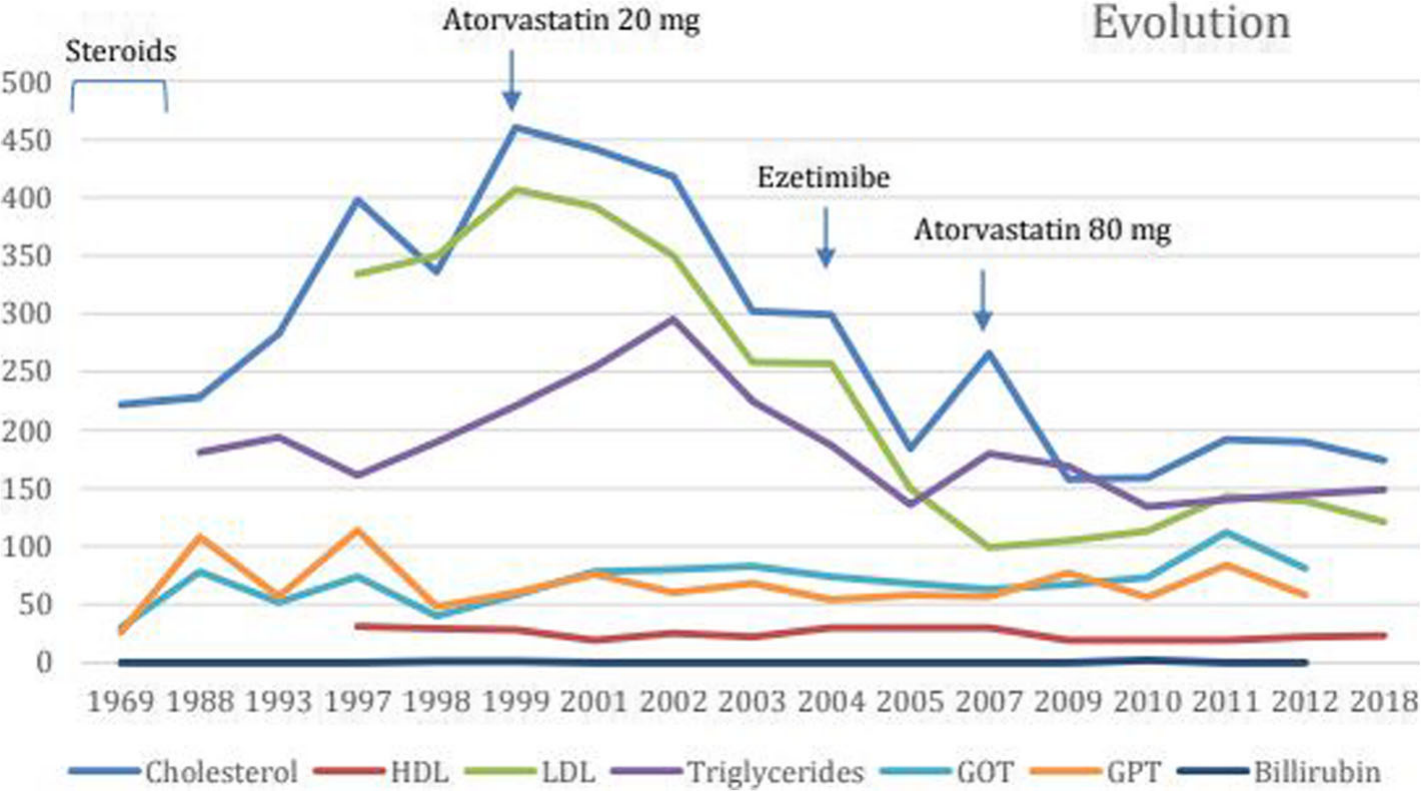
Lipid profile evolution shows a significant reduction of lipid concentrations (almost 70% of total cholesterol and LDL reduction). LDL: low-density lipoproteins, HDL- high-density lipoproteins, GOT: glutamic oxaloacetic transaminase, GPT: glutamic pyruvic transaminase

The patient continues regular follow-ups and is currently 55. She leads an active life despite mild dyspnoea. She is not receiving any specific treatment for NPD, but high doses of atorvastatin (80 mg) and ezetimibe have been administered for the past 10 years, without alterations in transaminases nor muscle aches. Her current lipid profile values are total cholesterol 174 mg/dL, low-density lipoproteins (LDL) cholesterol 121 mg/dL, HDL cholesterol 23 mg/dL and triglycerides 149 mg/dL (Fig. 4**)**.

## Discussion and conclusions

The result of a genetic test showed that our patient was double heterozygote for *SMPD1*: NM_000543.4:c.[84delC];[96G > A]. The C deletion at position 84 of cDNA promotes a frameshift resulting in an early stop codon, what presumably generates a null allele [NP_000534.3:p.(Gly29AspfsTer48)]. This variation was first described by Sikora *et al* [5] in 2003 in an double heterozygote Dutch child, being the second mutated allele NM_000543.4:c.748A > C (NP_000534.3:p.Ser250Arg) which causes null enzymatic activity [6]. The child died at the age of 5 years with a mild NPD type A phenotype and hepatosplenic enlargement, psychomotor delay and presence of foam cells in the bone marrow.

The switch of G to A at position 96 of cDNA induces a premature stop codon before the second in-frame initiation codon (NP_000534.3:p.Trp32Ter). This variation was first described by Pittis *et al* [7]. It is the most frequent mutation in Italian NPD type B patients (18.8% of the alleles). It has been described in 5 Italian patients (1 homozygous patient and 4 heterozygous presenting a second associated mutation, different from the one harboured by our patient). All 5 presented a NPD type B phenotype without neurological involvement regardless the associated mutation, with the exception of a 12-year-old heterozygous girl who developed symptoms during follow-up. All cases had organomegalies and 2 cases presented pulmonary involvement. This variation generates a premature stop codon that presumably would generate a truncated protein and thus should be considered as a null allele, expecting a severe phenotype. In fact, the in vitro functional characterization of the mutation through the expression of mutant SMPD1 in COS-1 cells confirms this hypothesis as nor the expression of protein neither residual enzymatic activity were detected [8]. However, this mutation is associated with a NPD type B phenotype, and residual enzymatic activity in leukocytes and fibroblasts is detected in patients, achieving 20.9% in the leukocytes of a homozygous patient [7], which is a high residual enzymatic value for a mutated protein. The reason of this discrepancy between the in vitro and the in vivo results is unclear, though may be related with differences in the post-translational modification and subcellular trafficking processes.

The SMPD1 gene has the peculiarity of having two in-frame initiation ATG codons at positions 1 and 33 and both can be functional in vitro [9]. Although in vivo translation of wild type SMPD1 initiates from the first initiation codon, the second codon can be functional when the first one has been disabled [7]. To become a mature and functional enzyme, ASM requires several post-translational modifications as well as a proper cellular trafficking [10]. In vitro expression of a mutant ASM lacking the signal peptide results in production of a non-glycosylated, non-secreted, cytosolic protein that lacks enzyme activity in cellular extracts [11]. When the second initiation codon substitutes the first one, the resulting protein misses 32 N-terminal residues of the signal peptide (which comprises 46 residues), so this variant most likely undergoes an abnormal maturing process. Therefore, the dysfunction is more likely to be related to gene expression, maturation and intracellular trafficking than to catalytic activity per se of the enzyme, as is often the case when pathogenic mutations are considered.

In our case, both mutations are located before the second initiation codon and both mutations would result in a null allele if the second codon were not functional, as they introduce early stop codons. Although the underlying mechanism is not fully understood, it is clear that none of the mutations seem to result in a null allele and that the second codon is, at least partially, activated in both alleles. The enzymatic activity of ASM is reduced enough to generate symptoms, but at the same time is high enough to be compatible with a NPD type B phenotype.

This is a unique case with a compound heterozygosity with mutations that induce early stop codons and are located in the same region before the second initiation codon, a circumstance that has not been previously reported in the literature.

The correlation between genotype and phenotype in compound heterozygotes is not evident. Although an equivalent expression of both alleles should normally be expected, due to epigenetic factors and methylation conditions, the maternal inherited allele of the SMPD1 gene may be expressed preferentially [6]. In our case, although we have the family pedigree, we cannot draw any conclusion from this matter since we cannot derive from our data the contribution of each mutation individually and theoretically a similar behaviour may be expected.

NPD can pose a diagnostic challenge. Differential diagnosis includes Wilson disease, Leigh syndrome, adrenoleukodystrophy, arginase deficiency and Gaucher disease. In LAL deficiency, the clinical spectrum can be similar to NPD, including the lipid profile alteration, but lung involvement is not commonly reported in LAL deficiency patients [12]. In Gaucher disease, total cholesterol, as well as LDL and HDL cholesterol are significantly reduced [13]. The lipid profile of our patient led to the suspicion that the initial diagnosis of Gaucher disease was incorrect. However, some authors describe an association between a low HDL-C level and defects in the SMPD1 gene causing Type B NPD [14]. The pathogenesis of the dyslipoproteinemia in these patients is not yet fully understood.

There are no recommendations for the lipid-lowering management in NPD. Some authors report that statins or fibrates may increase transaminases without a substantial reduction of lipid concentrations in NPD type B patients [15, 16]. However, there are cases describing an improvement of lipid profiles and reduction of liver enzymes under treatment with fenofibrate [15]. In our case, our patient received atorvastatin 80 mg and ezetimibe for many years, with a significant reduction of lipid concentrations (almost 70% of total cholesterol and LDL reduction) and liver enzymes. Olipudase alfa, a recombinant human acid sphingomyelinase (ASM), is used as an enzyme replacement therapy for the treatment of non-neurologic manifestations of acid sphingomyelinase deficiency. A 30-month follow-up clinical trial showed statistical significant reductions in liver (31%) and spleen (39%) volumes [17]. There was a mean increase in lung diffusing capacity of 35%, and clinically relevant improvements in infiltrative lung disease parameters, and lipid profiles improved in all 5 patients. Improvements in bone mineral density of the spine were observed in some patients. Lyso-sphingomyelin in dried blood spots decreased with olipudase alfa treatment [17]. We offered our patient to be treated with olipudase alfa, sending her to another hospital to be included in a clinical trial, but she repeatedly refused.

In conclusion, we present a case of NPD type B with a unique genetic mechanism: a compound heterozygosity of mutations that induce a premature stop codon located before the second initiation codon, circumstance that has not been reported in the literature. We also discuss the pharmacological management of the lipid profile, showing that, in our patient, a combination of a high intensity statin with ezetimibe was useful, although more research is needed to broaden the available evidence on this topic.

## Data Availability

All material is available if required.

## Abbreviations

NPD: Niemann-Pick disease
CNS: Central nervous system
BM: Bone marrow
ASM: Acid sphingomyelinase.
HDL: High-density lipoproteins
CT: Computerized tomography
LDL: Low density lipoproteins
